# Flexible bronchoscopy and mechanical ventilation in managing Mounier-Kuhn syndrome: a case report: Broncoscopia flexível e ventilação mecânica no tratamento da síndrome de Mounier-Kuhn: relato de caso

**DOI:** 10.1590/1516-3180.20160336270117

**Published:** 2017-04-20

**Authors:** Aslihan Gürün Kaya, Aydin Çiledağ, Çetin Atasoy, Demet Karnak

**Affiliations:** I MD. Professor, Department of Chest Diseases, Ankara University School of Medicine, Ankara, Turkey.; II MD. Professor, Department of Radiology, Ankara University School of Medicine, Ankara, Turkey.

**Keywords:** Bronchoscopy, Respiration, artificial, Optical fibers, Tracheobronchomegaly, Positive-pressure respiration

## Abstract

**CONTEXT:**

Mounier-Kuhn syndrome is a rare congenital condition with distinct dilatation and diverticulation of the tracheal wall. The symptoms may vary and the treatment usually consists of support.

**CASE REPORT:**

The patient was a 60-year-old male with recurrent hospital admission. He was admitted in this case due to dyspnea, cough and sputum production. An arterial blood sample revealed decompensated respiratory acidosis with moderate hypoxemia. A chest computed tomography (CT) scan showed dilatation of the trachea and bronchi, tracheal diverticula and bronchiectasis. Flexible bronchoscopy was performed, which revealed enlarged airways with expiratory collapse. Furthermore, orifices of tracheal diverticulosis were also detected. Non-invasive positive pressure ventilation (NPPV) was added, along with long-term oxygen therapy. At control visits, the patient’s clinical and laboratory findings were found to have improved.

**CONCLUSION:**

Flexible bronchoscopy can be advocated for establishing the diagnosis and non-invasive mechanical ventilation can be used with a high success rate, for clinical wellbeing in Mounier-Kuhn syndrome.

## INTRODUCTION

Mounier-Kuhn syndrome is a rare congenital condition characterized by distinct dilatation, and often by diverticulation of the trachea and central bronchi, in association with thinning or atrophy of the elastic tissue.^1^^,^^2^ The diagnosis is established through radiological and bronchoscopic findings. The treatment usually consists of support (Table 1).^3^^,^^4^

**Table 1.** Search of the literature in medical databases for case reports on Mounier-Kuhn syndrome. The search was last updated on February 3, 2017

Database	Search strategies	Papers found	Related papers
MEDLINE (via PubMed)	(Mounier Kuhn [Title]) AND tracheobronchomegaly [Title]	42	36
MEDLINE (via PubMed)	(Mounier Kuhn [Title]) AND tracheobronchomegaly [Title]) case reports [Publication Type]	31	29
Embase (via Elsevier)	(Mounier Kuhn [Title]) AND tracheobronchomegaly [Title]) case reports [Publication Type]	6	5
LILACS (via Bireme)	(Mounier Kuhn [Title]) AND tracheobronchomegaly [Title]) case reports [Publication Type]	1	1

We report a male case of Mounier-Kuhn syndrome that was diagnosed through observation of orifices of tracheal diverticula and tracheobronchomegaly, using flexible bronchoscopy (FB). The case was successfully treated by means of non-invasive mechanical ventilation (NIMV).

## CASE REPORT

A 60-year-old cachectic male was admitted to our hospital with complaints of dyspnea and cough. The patient had previously been diagnosed with chronic obstructive pulmonary disease (COPD), bronchiectasis and bullous emphysema, approximately 10 years earlier in another hospital. On that occasion, he had been treated with long-acting beta agonist, long-acting muscarinic antagonist and inhaler corticosteroid. He had a 20 pack-year smoking history and, for years, he had had recurrent admissions with pulmonary tract infections. Physical examination revealed decreased breathing sounds, across both sides of the lungs. Inspiratory coarse crackles were observed with finger-clubbing changes.

**Figure 1.**
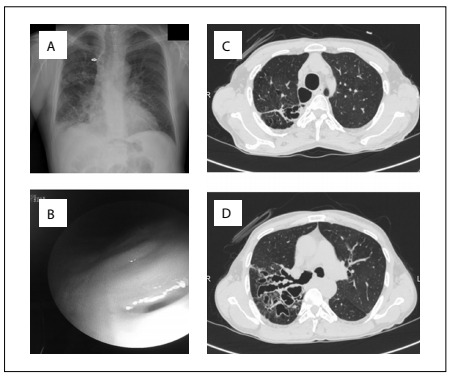
A. Chest X-ray showing ill-defined opaque areas, reticular dense areas and cystic lesions, predominantly in the right middle and lower zone; and also right paratracheal lucency (arrow). B. Diverticulum orifices on the tracheal wall seen in bronchoscopy. C and D. Chest computed tomography showing dilated trachea and bilateral main stem bronchi diverticula arising from the intrathoracic trachea; bilateral cystic bronchiectasis presenting predominantly in the right middle and lower lobe.

A chest X-ray showed right paratracheal lucency, ill-defined opaque areas, reticular dense areas and cystic lesions predominantly in the right middle and lower zones (Figure 1A). Pulmonary function testing revealed a mainly restrictive but also obstructive pattern with forced expiratory volume in one sec (FEV1) of 2.01 liters (53%), forced vital capacity (FVC) of 2.49 liters (59%) and FEV1/FVC of 88%. Arterial blood gases showed hypoxemia and hypercapnia with respiratory acidosis (pH 7.30; PaO_2_ 50 mmHg; PaCO_2_ 69 mmHg; HCO_3_ 28.3 mmol/l; and SaO_2_ 82%).

Administration of nasal oxygen at 2 liters/min and NIMV were started. The inspiratory and expiratory pressures were started as 15/5 mmHg and then were titrated to 20/5 mmHg. ST (spontaneous/timed) mode was selected because of the patient’s insufficiency of inspiratory effort. Over the next few days, oxygenation improved and this revealed compensated respiratory acidosis with NIMV (pH 7.38; PaO_2_ 61 mmHg; PaCO_2_ 55 mmHg; HCO_3_ 27.5 mmol/l; and SaO_2_ 92%), which probably reflected improvements in tidal volume and reduction in airway expiratory collapse. Diverticulum orifices on the lateral tracheal wall (Figure 1B) and tracheal/main stem bronchial enlargement were noted, with expiratory collapse of the airways, seen using flexible bronchoscopy. Bronchial brushing was performed on the right upper lobe posterior segment and right lower lobe apical segment. After specimen collection, bronchial lavage was performed on both sides using n-acetyl cysteine solution to facilitate expectoration, also known as bronchial toilet. The cytological examination of these materials was negative for malignancy and/or acid-fast bacilli (AFB) staining. Common bacterial and AFB cultures were also negative.

Chest computed tomography was also performed. This demonstrated abnormally dilated trachea, with bilateral main stem bronchi and two diverticula arising from the intrathoracic trachea. Bilateral cystic bronchiectasis was present predominantly in the right middle and lower lobe and mosaic attenuation was observed in lung fields bilaterally (Table 2 and Figures 1C and D).

**Table 2.** Patients’ airway diameters used for establishing the diagnosis of Mounier-Kuhn syndrome^1^

	Trachea (subcarinal junction)	Trachea (midsternum)	Carina	Right main bronchus	Left main bronchus
Transverse diameter (cm)	2.6* (2.7)^†^	2.6* (2.9)^†^ (3.4)^‡^ (5.0)^§^	2.1* (3.2)^†^	1.5* (2.2)^†^ (2.9)^‡^ (3.8)^§^	1.5* (1.8)^†^ (2.8)^‡^ (3.4)^§^
Antero-posterior diameter (cm)	2.5* (2.5)^†^	2.6* (2.7)^†^	2.9* (2.7)^†^	-	-

*Shah et al.^1^; ^†^Our patient’s diameters are shown in parentheses. Some additional patient diameters are also shown in parentheses, from ^‡^Celik et al.^3^; ^§^Abdelghani et al.^4^

Mucolytic treatment and physical rehabilitation therapy including postural drainage were arranged. Long-term oxygen and NIMV treatment were planned for home management. Pneumococcal and influenza vaccination were also suggested, to avoid recurrent infections. Regular follow-up visits were planned. The patient was discharged and has been doing well, with oxygen saturation increased to 89%. Furthermore, the patient has not had any serious complaints again, at follow-up visits.

## DISCUSSION

Mounier-Kuhn syndrome (MKS) is a rare congenital condition characterized by dilatation of airways, tracheal diverticulosis and bronchiectasis, associated with thinning or atrophy of the elastic tissues. Numerous diverticula between the tracheal cartilages and bulging dilatations in the posterior wall of the trachea may be present and are characteristic of MKS. It is assumed that these are non-muscular segments protruding between the tracheal cartilages. Histological findings of enlarged airways were described in 1987, but the first clinical description was made in 1932. The etiology of these findings remained uncertain, but congenital atrophy of smooth muscle and elastic tissue of the trachea and main bronchi was observed. Dilatation of the trachea and proximal bronchi causes impaired clearance of secretions, inefficient cough, persistent airway inflammation and subsequent distal bronchiectasis and/or emphysema.^1^^,^^2^ In the evaluation on the patient of the present report, no assessment of enlargement of the airways was performed at the time of his COPD/bronchiectasis diagnosis.

To date, the diagnosis of MKS is purely radiological. If the transverse diameter of the trachea exceeds 3.0 cm, right bronchus 2.4 cm and left bronchus 2.3 cm, the diagnosis is established.^3^ Other tracheal size thresholds include 2.5 cm for men and 2.1 cm for women.^1^ Radiological findings need to be supported by clinical findings. Radiologically, MKS is manifested not only through dilatation of the tracheobronchial system, but also through protrusion of the redundant musculomembranous tissue between the cartilaginous rings.^3^^,^^4^

MKS has three subtypes. In type 1, there is slight symmetrical dilation of the trachea and main bronchi. In type 2, the dilation and diverticula are distinct. In type 3, diverticular and saccular structures extend to the distal bronchi.^2^^,^^3^^,^^4^ Our patient fitted well into the third subtype, with presence of saccular changes away from the trachea (Table 2).

MKS is frequently seen in middle-aged men, and most of the patients are smokers.^1^^,^^2^ Most of the cases are sporadic, but familial cases have been described, with possible recessive inheritance.^5^ This patient was a smoker but had no familial history of cough or excessive sputum among his family members, or any consanguineous marriage.

During flexible bronchoscopy, the increased tracheal and main bronchial diameter can be detected.^1^^,^^2^ These semicircular folds of mucous membrane with saccular pouches have been described as tracheal or bronchial diverticulosis. Flexible bronchoscopy may easily detect such diverticula. Also, dynamic tracheal collapse may be viewed by means of bronchoscopy and is considered to be the gold standard for diagnosing tracheomalacia.^6^ Bronchoscopy is important not only for making the diagnosis, but also for enabling treatment options such as airway stenting.^2^^,^^7^

In our patient, the diagnosis of MKS was strongly supported by detection of diverticulum orifices and enlargement of the trachea and main stem bronchi, seen on flexible bronchoscopy. Thus, in this patient, it was important to detect tracheobronchomegaly, rather than bronchiectasis, especially for the course of the management.

The treatment options are usually symptomatic. Mucolytic therapy and physiotherapy have been used to increase separation of the sputum and facilitate expectoration.^2^ Lung transplantation has been reported.^1^ Expiratory airway collapse may cause serious breathing problems. NIMV has shown promising results in reducing symptoms in several cases, through serving to decrease pulmonary resistance and respiratory work load and improve expiratory flow and symptom control.^2^^,^^6^^,^^7^ In our patient, we used bi-level positive airway pressure to manage both expiratory airway collapse and hypercapnic respiratory failure.

## CONCLUSION

Flexible bronchoscopy can be advocated for establishing the diagnosis of Mounier-Kuhn syndrome and NIMV can be used with a high success rate for clinical wellbeing in cases of this syndrome.
